# Cognitive Impairments in Fibromyalgia Syndrome: Associations With Positive and Negative Affect, Alexithymia, Pain Catastrophizing and Self-Esteem

**DOI:** 10.3389/fpsyg.2018.00377

**Published:** 2018-03-22

**Authors:** Carmen M. Galvez-Sánchez, Gustavo A. Reyes del Paso, Stefan Duschek

**Affiliations:** ^1^Department of Psychology, University of Jaén, Jaén, Spain; ^2^Department of Psychology, University for Health Sciences, Medical Informatics and Technology, Hall in Tirol, Austria

**Keywords:** fibromyalgia, chronic pain, cognitive impairment, affective regulation, alexithymia, pain catastrophizing, self-esteem

## Abstract

Fibromyalgia syndrome (FMS) is a chronic condition characterized by widespread pain accompanied by symptoms like depression, anxiety, sleep disturbance and fatigue. In addition, affected patients frequently report cognitive disruption such as forgetfulness, concentration difficulties or mental slowness. Though cognitive deficits in FMS have been confirmed in various studies, not much is known about the mechanisms involved in their origin. This study aimed to investigate the contribution of affect-related variables to cognitive impairments in FMS. For this purpose, 67 female FMS patients and 32 healthy control subjects completed a battery of cognitive tests measuring processing speed, attention, visuospatial and verbal memory, cognitive flexibility and planning abilities. In addition, participants completed self-report questionnaires pertaining to positive and negative affect, alexithymia, pain catastrophizing and self-esteem. Clinical characteristics including pain severity, symptoms of depression and anxiety, insomnia and fatigue were also assessed. FMS patients showed markedly poorer performance than healthy controls in all of the cognitive domains assessed, in addition to greater levels of depression, anxiety, negative affect, alexithymia and pain catastrophizing, and lower self-esteem and positive affect. In exploratory correlation analysis in the FMS sample, lower cognitive performance was associated with higher pain severity, depression, anxiety, negative affect, alexithymia and pain catastrophizing, as well as lower self-esteem and positive affect. However, in regression analyses, pain, self-esteem, alexithymia, and pain catastrophizing explained the largest portion of the variance in performance. While interference effects of clinical pain in cognition have been previously described, the present findings suggest that affective factors also substantially contribute to the genesis of cognitive impairments. They support the notion that affective disturbances form a crucial aspect of FMS pathology, whereas strategies aiming to improve emotional regulation may be a beneficial element of psychological therapy in the management of FMS.

## Introduction

Fibromyalgia syndrome (FMS) is a chronic disorder characterized by widespread musculoskeletal pain and accompanying symptoms like depression, anxiety, sleep disturbance and fatigue. Its prevalence is estimated at 2–4% in the general population, with women being predominantly affected (Wolfe et al., 2010). FMS is associated with a severe reduction of quality of life and psychosocial impairments (Arnold et al., 2008). While not much is known about the etiology of FMS, current models assume a key role of sensitization of central nociceptive pathways in pain genesis (Gracely and Ambrose, 2011).

Complaints about cognitive disruption, such as forgetfulness, concentration difficulties or mental slowness are also frequent in FMS patients. These impairments, also referred to as “fibro fog,” may significantly affect patients’ everyday life and therefore are perceived to be among the most serious symptoms of the disease (Katz et al., 2004; Glass et al., 2005; Arnold et al., 2008; Williams et al., 2011). Problems in the representation of other people’s mental states (i.e., theory of mind) constitute another source of distress (Di Tella et al., 2015). The presence of cognitive impairments has been confirmed in numerous controlled studies. FMS patients displayed lower performance than healthy controls in tasks measuring attention and memory functions (Dick et al., 2008; Duschek et al., 2013; Montoro et al., 2015; Bar-On Kalfon et al., 2016) and cognitive processing speed (Veldhuijzen et al., 2012; Cherry et al., 2014; Reyes del Paso et al., 2015; Bar-On Kalfon et al., 2016). Deficits have also been documented in higher cognitive domains, including planning abilities (Cherry et al., 2014), decision making (Walteros et al., 2011), abstract thinking (Verdejo-García et al., 2009), cognitive flexibility (Gelonch et al., 2016), arithmetic processing (Reyes del Paso et al., 2012) and language-related skills (Park et al., 2001; Leavitt and Katz, 2008; Bennett et al., 2009).

Though the occurrence of cognitive impairments in FMS has been well-established, not much is known about the mechanism involved in their origin. Interference effects of nociception and pain have been considered a relevant factor, as supported by studies showing positive correlations between the severity of clinical pain and the magnitude of cognitive decline (Munguía-Izquierdo et al., 2008; Reyes del Paso et al., 2012; Duschek et al., 2013; Weiss et al., 2013; Montoro et al., 2015). Pain is an attention-demanding stimulus that recruits brain areas also crucial in cognition, thereby reducing available cognitive processing resources (Reyes del Paso et al., 2012, 2015; Weiss et al., 2013). Affective symptoms of FMS may be additionally involved in the mediation of cognitive deficits (Reyes del Paso et al., 2012). Theoretical models and empirical observations suggest negative impact of aversive mood states on cognition (Eysenck et al., 2007; Snyder, 2013). Accordingly, various studies demonstrated stronger cognitive impairments in FMS patients exhibiting more severe symptoms of depression and anxiety (Hassett et al., 2008; Munguía-Izquierdo et al., 2008; Gelonch et al., 2016, 2017). However, some FMS studies directly comparing the impacts of depression and anxiety with the impact of pain severity revealed the closest connection between pain and performance (Reyes del Paso et al., 2012, 2015; Duschek et al., 2013; Weiss et al., 2013). As such, the extent of the contribution of affective symptoms to cognitive decline cannot yet be determined with certainty.

While most available FMS studies on the implications of affect-related variables in cognitive impairments refer to symptoms of depression and anxiety, mood states (i.e., positive and negative affect) have not yet been specifically addressed. Positive and negative affect are regarded as virtually independent dimensions, where positive affect implies enthusiasm, activity and vitality-energy, while negative affect constitutes a general state of distress including aversive emotions like anger, fear and guilt (Watson et al., 1988). There is evidence of cognitive facilitation during positive affect and disruption of cognitive performance due to negative affect (Spering et al., 2005; Brand et al., 2007; Mitchell and Phillips, 2007; Rowe et al., 2007). FMS patients are assumed to experience emotional imbalance, i.e., increased negative and reduced positive affect (Hassett et al., 2008; Finan et al., 2009; Malin and Littlejohn, 2013); as such, it seems plausible that unfavorable affective states may also contribute to their cognitive impairments.

Affect-related coping styles may also be of interest in the present context. Alexithymia is conceptualized as a personality trait related to deficits in the cognitive processing of emotions that involves a lack of emotional awareness, difficulties in identifying and communicating feelings and an externally oriented thinking style (Bagby et al., 1994a, 2006). Several studies revealed markedly increased levels of alexithymia in FMS and its expression is associated with pain severity, disability and reduced quality of life (Castelli et al., 2012; Martínez et al., 2014; Montoro et al., 2016). In addition, the maladaptive coping strategy of pain catastrophizing is believed to play a key role in FMS pathology (Gracely et al., 2004; Baastrup et al., 2016). Pain catastrophizing, defined as an exaggerated negative orientation to pain, is an important source of fear and discomfort, which in turn may increase pain perception (Severeijns et al., 2002). It has been argued that both alexithymia and pain catastrophizing can interfere with optimal attentional processing and therefore may contribute to impairments in higher cognitive function (Crombez et al., 1998; Schütze et al., 2010; Montoro et al., 2016; Keefe et al., 2017).

Finally, self-esteem may be another relevant aspect. Self-esteem is a component of self-concept defined as an individual’s set of thoughts and feelings about his or her own worth and importance, resulting in a global positive or negative attitude toward oneself (Rosenberg, 1965). There is evidence of reductions in self-esteem and self-efficacy in FMS patients (Michielsen et al., 2006; Miró et al., 2011b; Garaigordobil, 2013; Peñacoba-Puente et al., 2015). Positive self-esteem is related to greater self-control and self-confidence (Michielsen et al., 2006; Garaigordobil, 2013), where self-esteem contributes to an individual’s intrinsic motivation, facilitating success in cognitive tasks (Zafra-Polo et al., 2014). Lack of self-esteem may thus constitute another possible affective feature involved in cognitive decline in FMS.

In sum, affect-related factors, including affective dysbalance, alexithymia, pain catastrophizing and low self-esteem, may contribute to cognitive impairments in FMS, in addition to pain experience and symptoms of emotional disorders. However, knowledge concerning the actual magnitude of their impacts remains scarce. Therefore, the main objective of the present study was to evaluate the relationship between self-report measures of these factors and performance on a comprehensive battery of cognitive tests measuring processing speed, attention, visuospatial and verbal memory, and cognitive flexibility, as well as mental planning and organizational skills. Clinical characteristics including pain severity, symptoms of depression and anxiety, as well as sleep disturbance and fatigue were regarded as additional predictors. Moreover, in order to quantify the magnitude of the cognitive decline in the investigated FMS patients, their test performance was compared with a control group of healthy individuals matched for relevant sociodemographic variables.

The following hypotheses were tested in the study: (1) Positive affect is positively, and negative affect is inversely, related with cognitive performance in FMS patients. (2) Higher levels of alexithymia and pain catastrophizing, and reduced self-esteem, predict lower performance. (3) Clinical pain severity, symptoms of depression and anxiety, as well as insomnia and fatigue demonstrate inverse associations with test performance. (4) In addition, FMS patients perform poorer than healthy individuals on the applied cognitive tasks.

## Materials and Methods

### Participants

In total, 67 women with FMS, recruited from the Fibromyalgia Association of Jaén (Spain), participated in the study. All of them were examined by a rheumatologist and met the 1990 American College of Rheumatology criteria for FMS (Wolfe et al., 1990). The control group comprised 32 healthy women. As the main research objective of the study pertained to the analysis of relationships between affect-related variables and cognitive performance within the FMS group, a smaller control group seemed sufficient. Exclusion criteria for both study groups included the presence of metabolic abnormalities, neurological disorders, drug abuse, and severe somatic (e.g., cancer) or psychiatric (e.g., psychotic) diseases. Control subjects were furthermore required not to suffer from acute or chronic pain of any kind. All participants were right handed.

### Cognitive Assessment

The *Rey-Osterrieth Complex Figure Test (ROCF)* (Rey, 1964; Spanish version by Peña-Casanova, 2009) was used to assess visuospatial memory performance. In the task, an abstract figure comprising 18 parts is presented and the participant has to copy it on a sheet of paper. Thirty minutes after this, he/she has to reproduce the figure from memory. The total number of correctly reproduced parts and execution time were taken as performance indices.

The *Verbal Learning Test (TAVEC)* (Benedet et al., 1998) was used to quantify verbal memory function. At the beginning of the test, a list of 16 words (shopping list) is read to the participant five times (List A); the participant has to reproduce as many words as possible directly after each trial (immediate free recall). Thereafter, another list is read once (List B) and then has to be reproduced (interference control condition). Following a 20 min break, the words of List A have to be reproduced again (long-delay recall). Thereafter, a list of 44 words is read, which includes all words of List A, some words of List B and further distractor words included in neither list A nor list B. The participant has to decide whether or not each of these words is part of List A (recognition). In the analysis, the number of correct responses during immediate free recall (List A and List B) and the number of correct words, omission errors (OE) and false positive responses (FP) during the recognition task were used as performance parameters. In addition, a discrimination score (DS) – as part of the recognition task – was computed according to the formula

DS=1−[(FP+OE)/44]×100.

Planning and organizational abilities were estimated by the *Zoo Map Task* (ZMT) from the Behavioural Assessment of the Dysexecutive Syndrome (Wilson et al., 1996; Spanish adaptation by Vargas et al., 2009). In this test, the participant has to plan a route to visit 6 of 12 possible locations in a zoo. The ZMT has two parts: i.e., (1) a more demanding open situation, in which little information is provided that would help to generate an appropriate plan, and (2) a situation that involves simply following a concrete, externally imposed strategy. Execution time and mistakes in each part in addition to the total number of correct responses were used as performance indices.

The *Revised Strategy Application Test (R-SAT)* was applied as a measure of strategic planning and self-regulation (Birnboim, 2004). The task includes three simple activities, i.e., figure tracing, sentence copying and object numbering. Activities are presented in two different stacks of 120 items each. Items differ in terms of their size (large, small) and time requirements (brief, medium, long). A large item scores 0 points and a small item scores 100 points, where participants are instructed to obtain as many points as possible. Moreover, items in which a face is displayed have to be avoided. The items are intermixed; however, the number of brief items decreases progressively within both stacks. As the execution time of the task is restricted to 10 min, the most efficient strategy is to complete brief items rather than longer ones. Thus, the predisposition to complete items in the presented sequence has to be overcome. Performance was indexed by the number of correct answers (brief items) and mistakes (long items and faces).

The *Trail Making Test (TMT)* was used to evaluate processing speed, attention and cognitive flexibility. The version of Delis et al. (2001) (Spanish adaption by Ibor, 2005) was applied instead of the standard form (Partington and Leiter, 1949), which provides more comprehensive performance assessment, also including aspects of visual scanning and motor speed. The test, in which visual targets (numbers, letters) are presented on sheets of paper, includes the following tasks, all of which have to be executed as fast as possible: (1) visual scanning (cross out all number 3 s on a page with different numbers), (2) number sequence (connect the numbers 1–16 in sequential order), (3) letter sequence (connect the letters A to P in alphabetic order), (4) switching (connect numbers and letters in alternating order, i.e., 1, A, 2, B etc.), and (5) motor speed (trace a predefined path). In addition to execution time, the following kinds of mistakes were recorded: (1) sequence (connection of correct item with an incorrect one), (2) set loss (connection of items of different categories) and (3) time out (exceeding the time limit of 250 s).

### Psychological Assessment

The patients’ clinical history and demographic data were obtained in a semi-structured interview. The Structured Clinical Interview for Axis I Disorders of the Diagnostic and Statistical Manual for Mental Disorders (SCID, First et al., 1999) was applied to diagnose possible mental disorders. In addition, the following self-report questionnaires were administered (values of Cronbach’s α taken from the literature are indicated):

*McGill Pain Questionnaire (MPQ)* (Melzack, 1975; Spanish version by Lázaro et al., 1994). This 73-item instrument allows quantification of clinical pain severity. It includes the parameters of sensory pain (score range: 0–84), emotional pain (score range: 0–22) and total pain experience (score range: 0–146). In addition, current pain intensity is assessed via a 10 cm visual analogue scale (VAS). Values of Cronbach’s α between 0.56 (emotional pain) and 0.74 (total pain) were reported (Lázaro et al., 1994).

*State-Trait Anxiety Inventory (STAI)* (Spielberger et al., 1970; Spanish version by Spielberger et al., 1982). This instrument allows assessment of current and habitual anxiety levels (20 items for the State Anxiety and Trait Anxiety scales, respectively; 4-point Likert scales, score range: 0–60). Values of Cronbach’s α are 0.93 for the State Anxiety and 0.87 for Trait Anxiety scales (Spielberger et al., 1982).

*Beck Depression Inventory (BDI)* (Beck et al., 1961; Spanish adaptation by Sanz et al., 2003). This 21-item scale was applied to assess the severity of symptoms of depression (4-point Likert scales, scores range: 0–63). Cronbach’s α is 0.95.

*Fatigue Severity Scale (FSS)* (Krupp et al., 1989; Spanish version by Bulbena et al., 2000). This scale allows assessment of fatigue based on 9 items (7-point Likert scales, score range: 9–63). It has a Cronbach’s α of 0.88.

*Oviedo Quality of Sleep Questionnaire (COS)* (Bobes et al., 2000). The Insomnia subscale of this instrument, comprising of 9 items (5-point Likert scales, score range: 4–54), was used in the study. It was completed under interview. Cronbach’s α of the subscale is 0.88.

*Positive and Negative Affect Scale (PANAS)* (Watson et al., 1988; Spanish version by Sandín et al., 1999). This questionnaire comprises 20 items (adjectives), divided into two scales, i.e., positive and negative affect (5-point Likert scales, score range: 10–50). Values of Cronbach’s α are 0.92 for positive affect scale and 0.88 for negative affect scale.

*Toronto Alexithymia Scale (TAS-20)* (Bagby et al., 1994b; Spanish version by Martínez-Sánchez, 1996). This instrument measures alexithymia via the three subscales of Difficulty Identifying Feelings, Difficulty Describing Feelings and Externally Oriented Thinking. The global alexithymia score obtained by aggregation of the subscale scores was used in the study (5-point Likert scales, score range: 20–100, cut-off of global score 61). It means that from this score people can be diagnosed with alexithymia. Cronbach’s α of the global score is 0.76.

*Coping Strategies Questionnaire (CSQ)* (Rosenstiel and Keefe, 1983; Spanish version by Rodriguez et al., 2004). The 6-item Catastrophizing subscale of this instrument was applied to assess levels of pain catastrophizing (6 point Likert scales, score range: 0–36). Cronbach’s α is 0.89.

*Rosenberg Self-esteem scale (SS)* (Rosenberg, 1965; Spanish version by Martín-Albo et al., 2007). On this 10-item scale, higher values reflect higher self-worth and more positive feelings about the self (5-point Likert scales, score range: 10–40). Its Cronbach’s α is 0.84.

### Procedure

The study was conducted in two sessions performed on the same day. During the first session, a clinical psychologist took the patients’ clinical history, recorded sociodemographic data and medication use, and evaluated possible violations of the exclusionary criteria. After that, SCID interviews were conducted and the psychometric questionnaires presented. In the second session, the neuropsychological tests were administered in the following order: ROCF (copy), ZMT, R-SAT, ROCF (reproduction), TAVEC (free recall), TMT, and TAVEC (long-delay recall, recognition). The tests were presented in this order to avoid an interference effect of different cognitive domains, especially between visual and verbal memory. Between each test, participants had a break of 5 min. The study protocol was approved by the Ethics Committee for Human Research of the University of Jaén and all participants provided written informed consent.

### Statistical Analysis

The assumption of normal distribution of the variables was tested using the ratio of kurtosis and asymmetry to the standard error. In all variables, this ratio was in the range of -2 and +2, confirming that the normality assumption was not violated (DeCarlo, 1997; Ryu, 2011). Comparisons between FMS patients and healthy individuals in clinical and demographic variables were performed using independent samples *t*-tests and χ2-tests. Group differences in cognitive performance were analyzed by means of multivariate analysis of variance (MANOVA). Age, years of education and body mass index were entered as covariates in this analysis. Effects of medication use and comorbid depression and anxiety disorders were evaluated by stratified analyses done for the FMS group (MANOVA) that compared patients using and not using each type of medication (separately for antidepressants, anxiolytics, non-opioid analgesics, and opiates), and comparing patients suffering and not suffering from depression and anxiety disorders. Effect sizes are indicated by adjusted eta squared ($\eta_{p}^{2}$).

Associations between questionnaire scores and neuropsychological test results were evaluated in two steps, both restricted to the FMS group (*N* = 67). Firstly, at an exploratory level, Pearson correlations were computed. Secondly, multiple regression analyses were performed. Two blocks of variables were used as predictors in the analyses: (1) to control for the effects of age, body mass index and years of education, these variables were entered simultaneously (enter method); (2) to determine psychological predictors of cognitive performance, questionnaire scales that showed significant correlations with neuropsychological parameters in the exploratory analysis were included (stepwise method). SPSS software (version 19.0) was employed for data analysis (IBM Corporation, Armonk, NY, United States).

## Results

**Table 1** displays the demographic and clinical data of both study groups. The groups did not differ significantly in age or level of education. In the applied questionnaires, FMS patients reported more severe symptoms of depression, anxiety, insomnia and fatigue than controls. Elevated levels of alexithymia, pain catastrophizing and negative affect, and lower level of positive affect and self-esteem, were also observed in FMS patients. Concerning alexithymia, 53 (73%) FMS patients and 10 (45%) healthy participants exhibited TAS-20 values above the cut-off score.

**Table 1:** Sociodemographic and clinical variables and questionnaire scores in FMS patients (*N* = 67) and healthy individuals (*N* = 32) (M ± SD or number and %) and statistics of the group comparisons (Student’s *t*-tests or χ^2^-tests).

	FMS patients	Healthy individuals	*t* or χ^2^	*p*	$\eta_{p}^{2}$
Age	52.55 ± 7.76	51.12 ± 6.60	0.89	0.37	0.01
Body mass index	27.92 ± 4.96	26.05 ± 3.04	1.95	0.05	0.03
Education (years)	10.53 ± 4.45	12.00 ± 4.34	-1.53	0.12	0.04
Depression (%)	58 (86.56%)	5 (15.62%)	47.10	<0.001	0.63
Anxiety disorders (%)	63 (94.02%)	6 (18.75%)	58.10	<0.001	0.74
Antidepressant use (%)	56 (83.58%)	5 (15.62%)	42.28	<0.001	0.57
Anxiolytic use (%)	56 (83.58%)	5 (15.62%)	35.96	<0.001	0.40
Analgesic use (%)	53 (79.10%)	3 (9.37%)	49.52	<0.001	0.66
Opiate use (%)	34 (50.74%)	0 (0%)	24.73	<0.001	0.25
Positive affect (PANAS)	29.17 ± 9.34	36.37 ± 7.25	-3.84	<0.001	0.13
Negative affect (PANAS)	32.92 ± 8.40	26.21 ± 10.54	3.42	0.001	0.11
Alexithymia (TAS-20)	58.14 ± 21.75	39.43 ± 19.70	4.12	<0.001	0.24
Catastrophizing (CSQ)	24.80 ± 11.21	2.34 ± 7.44	10.28	<0.001	0.65
Self-esteem (SS)	24.67 ± 7.81	33.09 ± 4.99	-5.57	<0.001	0.27
Current pain intensity (MPQ)	7.10 ± 2.25	3.28 ± 2.28	7.83	<0.001	0.48
Sensory pain (MPQ)	50.31 ± 25.36	13.68 ± 17.49	7.27	<0.001	0.49
Emotional pain (MPQ)	11.81 ± 6.91	2.71 ± 431	6.82	<0.001	0.44
Total pain (MPQ)	79.09 ± 39.09	22.06 ± 30.60	7.69	<0.001	0.52
Trait anxiety (STAI)	52.61 ± 12.36	26.40 ± 16.62	8.79	<0.001	0.63
State anxiety (STAI)	23.59 ± 11.16	15.56 ± 11.64	3.29	<0.001	0.15
Depression (BDI)	43.22 ± 13.54	14.50 ± 17.49	8.96	<0.001	0.63
Fatigue (FSS)	52.50 ± 9.96	25.65 ± 17.04	9.81	<0.001	0.63
Insomnia (COS)	38.91 ± 11.34	14.81 ± 10.93	10.00	<0.001	0.74


*Anxiety disorders comprise panic disorder, generalized anxiety disorder, phobias and adjustment disorder.*

### Group Differences in Cognitive Performance

The MANOVA for the neuropsychological test scores showed a multivariate group effect (*F*[26, 72] = 3.11, *p* < 0.0001, $\eta_{p}^{2}$ = 0.53). **Table 2** displays the means and standard deviations of the performance indices, together with the statistics of the univariate group comparisons. The results for most test parameters reflect lower task speed and accuracy in FMS patients than controls. Differences were significant for all speed and accuracy parameters of the ROCF (copy and reproduction conditions) and TAVEC (immediate and long-delay free recall, and recognition). In the ZMT, group differences were seen in the total number of correct responses, execution time for both versions and mistakes in version 1. On the R-SAT, differences arose for the number of correct responses (short items). Among the parameters of the TMT, execution times for the number sequence, letter sequence, switching and motor speed tasks, as well as set loss and time out mistakes, differed between groups. The covariates of age, years of education and body mass index were non-significant. No differences were found between subgroups of patients taking and not taking antidepressants, anxiolytics, non-opioid analgesics, or opioids, or between patients suffering and not suffering from comorbid depression or anxiety disorders (all *F*s ≤ 3.18, all *p*s ≥ 0.06).

**Table 2:** Mean (±SD) of neuropsychological test scores of FMS patients (*N* = 67) and healthy individuals (*N* = 32) and statistics of the univariate group comparisons.

		FMS patients	Healthy individuals	*F*[4,64]	*p*	$\eta_{p}^{2}$
ROCF	Copy total correct marks	33.02 5.08	35.67 0.59	9.42	<0.001	0.28
	Reproduction total correct marks	14.76 6.14	23.35 9.14	9.74	<0.001	0.29
	Copy execution time	252.53 105.76	168.75 88.12	7.59	<0.001	0.24
	Reproduction execution time	155.82 70.11	121.87 78.42	5.03	<0.001	0.17

ZMT	Total correct responses	12.19 2.80	13.57 2.48	4.57	0.002	0.16
	Execution time version 1	239.35 125.39	179.06 82.44	6.29	<0.001	0.21
	Execution time version 2	158.47 105.71	90.12 50.32	6.84	<0.001	0.22
	Mistakes version 1	2.85 1.95	1.93 1.16	5.42	<0.001	0.18
	Mistakes version 2	0.85 1.04	0.46 0.84	2.81	0.10	0.03

TAVEC	List A (immediate free recall)	37.82 10.32	46.31 10.52	8.92	<0.001	0.27
	List B (interference control)	3.56 1.89	4.53 1.99	3.39	<0.001	0.12
	Long-delay recall	8.87 2.91	10.53 3.20	6.65	0.011	0.06
	Recognition correct responses	9.55 6.06	15.05 1.50	9.14	<0.001	0.28
	Recognition omission errors	6.43 6.03	0.84 1.50	9.14	<0.001	0.28
	Recognition false positives	16.23 18.15	3.06 4.91	4.43	0.002	0.15
	Discrimination score	33.08 59.04	81.24 28.83	8.83	<0.001	0.26

R-SAT	Correct responses (short items)	44.58 9.09	50.34 7.50	3.91	<0.001	0.14
	Mistakes long items	4.82 7.26	2.03 2.40	1.21	0.31	0.05
	Mistakes face items	1.58 2.06	0.43 0.91	0.06	0.07	0.09

TMT	Execution time 1 (visual scanning)	78.02 106.70	41.87 13.30	1.84	0.13	0.08
	Execution time 2 (number sequence)	96.01 63.59	60.31 25.75	10.07	<0.001	0.30
	Execution time 3 (letter sequence)	102.74 70.13	66.90 30.18	8.26	<0.001	0.26
	Execution time 4 (switching)	225.92 130.32	119.15 51.33	12.37	<0.001	0.34
	Execution time 5 (motor speed)	140.65 57.32	98.09 49.73	6.24	<0.001	0.21
	Sequence mistakes	2.47 3.59	0.90 1.51	1.73	0.15	0.07
	Set loss mistakes	2.94 5.54	0.31 0.69	3.23	0.002	0.12
	Time out mistakes	10.44 10.76	0.78 2.05	12.65	<0.001	0.35


*All execution times are indicated in s.*

### Correlations Between Clinical Variables and Cognitive Performance in FMS Patients

**Table 3** displays the correlations between clinical variables and the indices of cognitive test performance in FMS patients. Current pain intensity score (VAS) on the MPQ correlated negatively with the number of correct marks on both conditions of the ROCF and the correct responses in the recognition task of the TAVEC. This score correlated positively with execution time during the number sequence, letter sequence and motor speed tasks of the TMT, omission errors and false positive responses in the TAVEC recognition task, set loss and time out mistakes on the TMT, and execution time during version 2 of the ZMT. The sensorial and total pain scales correlated negatively with the number of correct responses on the ZMT, and positively with execution time during version 2 of this test. Emotional pain correlated negatively with correct responses on the TAVEC recognition task, and positively with omission errors and false positives. Trait anxiety (STAI) was positively associated with execution time during the copy condition of the ROCF, and with the visual scanning and motor speed tasks of the TMT. State anxiety was inversely associated with the number of correct responses (short items) on the R-SAT, and with the number of correct recognition responses, long-delay recall and the discrimination score of the TAVEC, and positively associated with long item mistakes on the R-SAT, omission errors in the TAVEC recognition task, and execution time during the TMT motor speed task. Depression (BDI) correlated positively with the number of long item mistakes on the R-SAT and set loss mistakes on the TMT, and negatively with correct responses on the R-SAT. Fatigue (FSS) correlated positively with execution time during the motor speed task of the TMS. No significant correlations arose for insomnia (COS).

**Table 3:** Correlations between clinical variables and neuropsychological test scores in FMS patients.

		Current pain (MPQ)	Sensory pain (MPQ)	Emotional pain (MPQ)	Total pain (MPQ)	Trait anxiety (STAI)	State anxiety (STAI)	Depression (BDI)	Fatigue (FSS)	Insomnia (COS)
ROCF	Copy total correct marks	-0.32+	-0.19	-0.04	-0.15	-0.21	-0.18	0.01	-0.10	0.04
	Reproduction total correct marks	-0.37*	-0.06	-0.09	-0.06	-0.17	-0.23	-0.07	-0.20	0.03
	Copy execution time	0.11	0.15	-0.06	0.10	0.31+	0.15	-0.02	0.12	-0.10
	Reproduction execution time	0.16	0.24	0.07	0.21	0.23	0.12	0.03	0.10	0.04

ZMT	Total correct responses	-0.06	-0.30+	0.05	-0.26+	-0.01	-0.09	-0.12	-0.10	0.03
	Execution time version 1	0.01	0.05	-0.20	-0.01	0.04	-0.01	-0.18	0.05	0.09
	Execution time version 2	0.35*	0.28+	0.15	0.29+	0.09	0.01	0.12	0.21	-0.01
	Mistakes version 1	-0.05	0.28+	-0.03	0.23	0.02	0.07	0.17	0.02	0.01
	Mistakes version 2	0.16	0.16	-0.08	0.16	-0.01	0.06	-0.04	0.22	-0.16

TAVEC	List A (immediate free recall)	-0.07	0.06	0.13	0.08	0.01	-0.15	-0.03	0.10	0.14
	List B (interference control)	-0.19	0.07	0.08	0.07	-0.17	-0.17	-0.14	-0.02	-0.01
	Long-delay recall	-0.16	-0.05	-0.01	-0.04	0.03	-0.26+	-0.11	0.05	0.02
	Recognition correct responses	-0.29+	-0.19	-0.33*	-0.22	-0.17	-0.25+	-0.15	-0.17	0.07
	Recognition omission errors	0.29+	0.19	0.33*	0.22	0.18	0.25+	0.15	0.17	-0.08
	Recognition false positives	0.26+	0.13	0.39*	0.19	0.22	0.21	0.16	0.24	-0.05
	Discrimination score	-0.19	-0.02	-0.01	-0.06	-0.21	-0.26+	-0.12	-0.07	0.13

R-SAT	Correct responses (short items)	-0.12	-0.09	-0.13	-0.14	-0.02	-0.27+	-0.27+	-0.14	0.03
	Mistakes long items	0.10	0.20	0.18	0.22	0.06	0.25+	0.26+	0.22	-0.07
	Mistakes face items	0.07	0.22	0.16	0.23	-0.05	0.15	0.17	0.22	-0.03

TMT	Execution time 1 (visual scanning)	0.07	0.06	0.23	0.06	0.27+	-0.08	0.17	0.16	-0.01
	Execution time 2 (number sequence)	0.30+	0.15	0.01	0.11	0.17	0.17	0.14	0.16	-0.09
	Execution time 3 (letter sequence)	0.34*	0.09	0.10	0.07	0.22	0.21	0.21	0.21	-0.08
	Execution time 4 (switching)	0.16	0.11	-0.02	0.06	0.19	0.05	0.05	0.23	-0.09
	Execution time 5 (motor speed)	0.25+	0.13	0.09	0.13	0.33*	0.32*	0.13	0.30+	-0.06
	Sequence mistakes	0.22	0.20	0.16	0.20	-0.04	0.11	0.21	0.09	0.03
	Set loss mistakes	0.30+	0.17	0.24	0.19	0.13	0.16	0.24+	0.19	-0.10
	Time out mistakes	0.25+	0.19	0.01	0.14	0.20	0.05	0.04	0.23	-0.05


*+*p* < 0.05, ^∗^*p* < 0.01, two-tailed testing.*

### Correlations Between Affective Variables and Cognitive Performance in FMS

**Table 4** includes the correlations of the questionnaire scores for positive and negative affect, alexithymia, pain catastrophizing and self-esteem with the indices of neuropsychological test performance in FMS patients. Positive affect (PANAS) was inversely associated with execution time during version 2 of the ZMT, omission errors and false positives on the TAVEC recognition task, execution time during the visual scanning, number sequence, letter sequence and switching tasks of the TMT, as well as set loss and time out mistakes on the latter instrument. Moreover, positive affect correlated positively with correct responses (short items) on the R-SAT, and recall (List B) and recognition performance on the TAVEC, as well as the discrimination score provided by this test. Negative affect (PANAS) correlated positively with face item mistakes on the R-SAT, false positives on the TAVEC recognition task, execution time during the visual scanning task of the TMT, and sequence and set loss mistakes. Negative affect correlated negatively with execution time during version 1 of the ZMT.

**Table 4:** Correlations of positive and negative affect, alexithymia, pain catastrophizing and self-esteem with neuropsychological test scores in FMS patients.

		Positive affect (PANAS)	Negative affect (PANAS)	Alexithymia (TAS-20)	Pain catastrophizing (CSQ)	Self-esteem (SS)
ROCF	Copy total correct marks	0.20	0.01	-0.32*	-0.06	0.26+
	Reproduction total correct marks	0.03	0.04	-0.08	0.02	0.14
	Copy execution time	-0.24	0.03	0.28+	0.07	-0.41*
	Reproduction execution time	-0.21	0.18	0.39*	0.28+	-0.28*

ZMT	Total correct responses	0.08	-0.10	-0.16	-0.1	0.22
	Execution time version 1	-0.10	-0.24+	0.08	0.01	-0.26+
	Execution time version 2	-0.39*	0.06	0.21	0.20	-0.45*
	Mistakes version 1	-0.03	0.11	0.18	0.07	0.17
	Mistakes version 2	-0.12	0.09	0.10	0.11	0.18

TAVEC	List A (immediate free recall)	0.13	0.08	-0.15	0.11	0.23
	List B (interference control)	0.26+	-0.17	-0.18	-0.04	0.32*
	Long-delay recall	0.02	0.04	-0.23	-0.03	0.22
	Recognition correct responses	0.31+	-0.20	-0.36*	-0.36*	0.43*
	Recognition omission errors	-0.31+	0.20	0.36*	0.35*	-0.43*
	Recognition false positives	-0.30+	0.27+	0.33*	0.38*	-0.39*
	Discrimination score	0.28+	-0.16	-0.31+	-0.14	0.49*

R-SAT	Correct responses (short items)	0.25+	-0.15	0.06	0.04	0.35*
	Mistakes long items	-0.18	0.22	0.21	0.31+	-0.21
	Mistakes face items	-0.12	0.27+	0.22	0.41*	-0.12

TMT	Execution time 1 (visual scanning)	-0.27+	0.28+	0.25+	0.24	-0.30+
	Execution time 2 (number sequence)	-0.30+	0.13	0.36*	0.27+	-0.44*
	Execution time 3 (letter sequence)	-0.35*	0.24	0.44*	0.36*	-0.40*
	Execution time 4 (switching)	-0.32*	0.21	0.41*	0.18	-0.35*
	Execution time 5 (motor speed)	-0.06	0.12	0.10	0.16	-0.44*
	Sequence mistakes	-0.22	0.25+	0.22	0.30+	-0.27+
	Set loss mistakes	-0.35*	0.28+	0.30+	0.33*	-0.39*
	Time out mistakes	-0.25+	0.12	0.42*	0.17	-0.31+


*+*p* < 0.05, ^∗^*p* < 0.01, two-tailed testing.*

Alexithymia (TAS-20) correlated negatively with the number of correct marks in the copy condition of the ROCF, and with correct recognition responses and the discrimination score of the TAVEC, and positively with execution time during the copy and reproduction conditions of the ROCF, the visual scanning, number sequence, latter sequence and switching tasks of the TMT, set loss and time out mistakes on the TMT, as well as omissions and false positives on the TAVEC recognition task.

Pain catastrophizing (CSQ) correlated negatively with the number of correct recognition responses on the TAVEC, and positively with execution time during the reproduction condition of the ROCF, the number and letter sequence conditions of the TMT, long item and face item mistakes on the R-SAT, sequence and set loss mistakes on the TMT, as well as omissions errors and false positives in the TAVEC recognition task.

Self-esteem (SS) was inversely associated with execution time during the copy and reproduction conditions of the ROCF, both versions of the ZMT, the visual scanning, number sequence, letter sequence, switching and motor speed conditions of the TMT, as well as set loss, sequence and time out mistakes on the TMT, and omissions and false positives in the TAVEC recognition task. Moreover, self-esteem correlated positively with the number of correct marks in the copy condition of the ROCF, correct responses (short items) on the R-SAT, and free recall performance (List B) and the discrimination score of the TAVEC.

### Results of Multiple Regression Analysis

Significant results of the multiple regression analyses for the prediction of performance parameters, after controlling for the effects of age, years of education and BMI, are presented in **Table 5**. Here, current pain intensity (VAS of MPQ) was inversely related to the number of correct marks in the copy and reproduction conditions of the ROCF and execution time during version 2 of the ZMT; and positively related to execution time during the number and letter sequence tasks of the TMT, and to set loss and time out mistakes on this test. The sensorial pain index was positively associated to mistakes in version 1 of the ZMT; emotional pain was inversely associated with correct responses, and positively with omission errors in the TAVEC recognition task. Depression (BDI) was positively associated with long item mistakes on the R-SAT. State anxiety (STAI) was positively associated with execution time during the motor speed task of the TMT and trait anxiety was positively associated with execution time during the copy condition of the ROCF.

**Table 5:** Significant results of the second block (step-wise method) of the multiple regression analysis for prediction of neuropsychological test scores by clinical and psychological variables in FMS patients.

		*M*	Predictors	β	*r*^2^	Δ*r*^2^	*F*	*t*	*p*
ROCF	Copy total correct marks	1	Current pain (MPQ)	-0.33	0.34	0.10	-3.13	10.06	0.003
	Reproduction total correct marks	1	Current pain (MPQ)	-0.35	0.15	0.13	-2.90	9.96	0.005
	Copy execution time	1	Self-esteem (SS)	-0.36	0.33	0.13	-3.47	13.05	0.001
		2	Self-esteem (SS)	-0.26	0.36	0.04	-2.26	4.03	0.027
			Trait anxiety (STAI)	0.24			2.12		0.038
	Reproduction execution time	1	Alexithymia (TAS-20)	0.36	0.37	0.12	3.48	12.13	0.001

ZMT	Total correct responses	1	Total pain (MPQ)	-0.29	0.25	0.08	-2.69	7.21	0.009
	Execution time version 2	1	Self-esteem (SS)	-0.43	0.28	0.16	-3.90	3.79	<0.001
		2	Self-esteem (SS)	-0.33	0.35	0.17	-2.86	15.23	0.006
			Current pain (MPQ)	0.31			2.70		0.009
	Mistakes version 1	1	Sensorial pain (MPQ)	0.27	0.22	0.07	2.47	6.08	0.017

R-SAT	Correct responses (short items)	1	Self-esteem (SS)	0.35	0.19	0.13	3.02	10.29	0.004
	Mistakes (long items)	1	Depression (BDI)	0.34	0.06	0.10	2.72	6.92	0.008
	Mistakes (face items)	1	Catastrophizing (CSQ)	0.42	0.13	0.17	3.57	12.75	0.001
	List B (interference control)	1	Self-esteem (SS)	0.32	0.15	0.15	7.29	2.70	0.009

TAVEC	Recognition false positives	1	Catastrophizing (CSQ)	0.39	0.13	0.16	3.34	12.27	0.001
		2	Catastrophizing (CSQ)	0.31	0.18	0.07	2.61	5.67	0.011
			Self-esteem (SS)	-0.28			-2.25		0.028
	Recognition omission errors	1	Emotional pain (MPQ)	0.43	0.22	0.16	3.62	13.13	0.001
		2	Emotional pain (MPQ)	0.32	0.25	0.05	2.53	4.07	0.014
			Self-esteem (SS)	-0.25			-2.01		0.048
	Recognition correct responses	1	Emotional pain (MPQ)	-0.43	0.22	0.16	-3.62	13.12	0.001
		2	Emotional pain (MPQ)	-0.32	0.26	0.05	-2.52	4.18	0.014
			Self-esteem (SS)	0.25			2.04		0.045
	Discrimination score	1	Self-esteem (SS)	0.44	0.27	0.19	3.99	17.26	<0.001

TMT	Execution time 1 (visual scanning)	1	Negative affect (PANAS)	0.32	0.09	0.10	2.68	7.21	0.009
	Execution time 2 (number sequence)	1	Current pain (MPQ)	0.40	0.42	0.14	4.05	15.99	<0.001
		2	Current pain (MPQ)	0.30	0.48	0.06	2.96	6.85	0.004
			Self-esteem (SS)	-0.29			-2.90		0.005
		3	Current pain (MPQ)	0.30	0.51	0.04	3.08	4.79	0.003
			Self-esteem (SS)	-0.28			-2.36		0.022
			Alexithymia (TAS-20)	0.20			2.09		0.041
	Execution time 3 (letter sequence)	1	Current pain (MPQ)	0.43	0.37	0.13	4.22	12.88	<0.001
		2	Current pain (MPQ)	0.41	0.48	0.12	4.37	14.06	<0.001
			Alexithymia (TAS-20)	0.36			3.73		<0.001
		1	Alexithymia (TAS-20)	0.38	0.41	0.13	3.73	13.92	<0.001
	Execution time 4 (switching)	2	Alexithymia (TAS-20)	0.28	0.42	0.05	2.62	5.74	0.010
			Positive affect (PANAS)	-0.24			-2.40		0.020
		1	Self-esteem (SS)	-0.38	0.22	0.13	-3.35	3.15	0.001
	Execution time 5 (motor speed)	2	Self-esteem (SS)	-31	0.26	0.10	-2.72	7.94	0.009
			State anxiety (STAI)	0.24			2.09		0.041
	Sequence mistakes	1	Catastrophizing (CSQ)	0.31	0.07	0.10	2.60	6.74	0.012
		1	Self-esteem (SS)	-0.41	0.16	0.06	-3.43	1.21	0.001
	Set loss mistakes	2	Self-esteem (SS)	-0.31	0.20	0.15	-2.54	11.74	0.014
			Current pain (MPQ)	0.26			2.09		0.041
	Time out mistakes	1	Alexithymia (TAS-20)	0.36	0.32	0.25	3.27	6.97	0.002
		2	Alexithymia (TAS-20)	0.33	0.39	0.15	3.23	7.72	0.002
			Current pain (MPQ)	0.29			2.81		0.007


*Model (M), standardized β, adjusted *r*^*2*^, change in *r*^*2*^ (Δ*r*^*2*^), *F, t*, and *p* are indicated. Results of the first block, which served to control for the effects of age, education and BMI are not reported.*

Positive affect (PANAS) was inversely related to execution time during the switching task, and negative affect was positively related to execution time during the visual scanning task of the TMT. Alexithymia (TAS-20) was positively associated with execution time during the reproduction condition of the ROCF and the number sequence, letter sequence and switching tasks of the TMT, and with time out mistakes on this instrument. Pain catastrophizing (CSQ) was positively associated with face item mistakes in the R-SAT, false positive responses in the TAVEC recognition task, and sequence mistakes on the TMT. Self-esteem (SS) was inversely related to execution time during the copy condition of the ROCF, version 2 of the ZMT and the number sequence and motor speed tasks of the TMT. It furthermore was inversely related to false positive responses, omission errors in the TAVEC recognition task and set loss mistakes on the TMT. Self-esteem was positively associated with correct responses (short items) on the R-SAT, correct responses in the free recall (List B) and recognition tasks of the TAVEC, and the discrimination score of the test.

## Discussion

This study revealed poorer performance in FMS patients vs. healthy individuals on neuropsychological tests of processing speed, attention, visuospatial and verbal memory, cognitive flexibility, as well as mental planning and organizational skills. According to an exploratory correlation analysis conducted in the patient group, test performance was associated with pain severity, symptoms of depression and anxiety, as well as affect-related variables including positive and negative affect, alexithymia, pain catastrophizing and self-esteem. In subsequent regression analyses, pain, self-esteem, alexithymia, and catastrophizing explained the overall largest portion of test score variance.

The cognitive performance reduction in FMS patients confirms previous findings. The longer execution times during the number sequence, letter sequence and switching tasks, and the higher number of set loss and time out mistakes on the TMT, are in line with observations of deficits in attentional functions and processing speed (Park et al., 2001; Veldhuijzen et al., 2012; Cherry et al., 2014; Miró et al., 2015; Reyes del Paso et al., 2015; Bar-On Kalfon et al., 2016). Patients’ higher error rate and longer execution time during the ROCF reflect visuospatial memory impairments; the group differences in all parameters of the TAVEC indicate reduced performance in the free recall and recognition of verbal material in patients. This is in accordance with previous reports of deficits in these memory domains in FMS (Park et al., 2001; Dick et al., 2008; Cherry et al., 2014; Bar-On Kalfon et al., 2016). Moreover, the lower number of correct responses and longer execution time during the ZMS, and the performance reduction in the R-SAT, point toward planning and organizational deficits in FMS. This supports the notion that higher cognitive, i.e., executive, functions are also affected in FMS (Park et al., 2001; Verdejo-García et al., 2009; Walteros et al., 2011; Gelonch et al., 2016).

In the applied questionnaires FMS patients reported more severe symptoms of depression, anxiety, insomnia and fatigue than controls, which constitute typical bodily and mental symptoms accompanying FMS pain (Wolfe et al., 2010). Elevated levels of alexithymia, pain catastrophizing and negative affect, and the reductions in positive affect and self-esteem, have also been previously observed (Michielsen et al., 2006; Hassett et al., 2008; Garaigordobil, 2013; Montoro et al., 2015, 2016; Baastrup et al., 2016). The latter results confirm the view of emotional impairment as a crucial aspect of FMS pathology (Weiss et al., 2013; Rosselló et al., 2015; Montoro et al., 2016).

As its main aim, this study investigated the impact of FMS symptoms and affect-related variables on cognitive performance. Among clinical symptoms, pain severity was most closely related to neuropsychological test scores. In correlation analysis, high scores on the MPQ scales were associated with lower speed and accuracy on the attentional and planning tasks (TMT and ZMT), as well as fewer correct responses and more mistakes on both memory tests (ROCF and TAVEC). Similar associations also arose in regression analysis. The associations between MPQ scale scores and cognitive function are in line with previous FMS studies, where they were interpreted as indicative of an interference effect of pain with cognitive processing (Reyes del Paso et al., 2012, 2015; Duschek et al., 2013; Weiss et al., 2013). Depression and anxiety symptoms showed smaller impacts on cognition than pain. According to correlation analysis, the BDI score correlated with correct responses and errors on the R-SAT, and with set loss mistakes on the TMT. Only the association with errors on the R-SAT also arose in regression analysis. Higher anxiety levels (STAI) were associated with lower performance speed on the visual scanning and motor speed tasks of the TMT, and the copy condition of the ROCF, and with less accurate performance on the R-SAT and TAVEC. In regression analysis, only the associations with motor speed (TMT) and performance in the ROCF copy task were seen. It is also important to note that in subgroup comparisons, cognitive performance did not differ between FMS patients diagnosed with comorbid depression and anxiety disorders and those not diagnosed with these clinical conditions. Moreover, fatigue only correlated positively with execution time during the motor speed task of the TMT, and insomnia was unrelated to performance parameters. Taken together, these results are consistent with previous FMS studies, according to which accompanying clinical symptoms, including those of depression, anxiety disorders, sleep disturbance and fatigue had a far smaller impact on cognitive performance than pain severity, suggesting that these factors only play a subordinate role (Dick et al., 2008; Verdejo-García et al., 2009; Reyes del Paso et al., 2012, 2015; Duschek et al., 2013; Weiss et al., 2013; Montoro et al., 2015).

The involvement of affect-related variables in cognitive impairment in FMS represents a novel aspect of this study. Positive affect demonstrated a close association with attentional and verbal memory performance. In correlation analysis, the corresponding PANAS scale score was predictive of faster performance and lower error rates in the visual scanning, number sequence, letter sequence and switching tasks of the TMT. This also applies to almost all TAVEC indices of verbal recall and recognition performance. A weaker association between positive affect and planning abilities is suggested by the negative correlation of the PANAS scale score with execution time during one of the two ZMT tasks, and by the positive correlation with correct responses on the R-SAT. Negative affect was related to more errors on the TMT, TAVEC and R-SAT and slower performance on one of the ZMT tasks. The associations of positive and negative affect with performance speed also arose in regression analysis. Beneficial effects of positive affect on cognition have repeatedly been reported in healthy individuals, for example in terms of facilitation of attentional processing, problem solving and decision making (Isen et al., 1987; Estrada et al., 1994; Isen, 2001; Rowe et al., 2007). Adverse effects of negative mood on performance are also well-known (Brand et al., 2007; Mitchell and Phillips, 2007; Hoffmann et al., 2017). According to our correlation analysis, positive affect was far more closely related to cognition than negative affect; as such, the lack of positive affect in FMS may play a particular role in the cognitive impairments experienced by patients.

Alexithymia also showed a close relationship with attentional and memory performance. The TAS-20 global score was positively related to execution time during the visual scanning, number sequence, letter sequence and switching tasks of the TMT, as well as to error rates in this test. It furthermore correlated negatively with speed and accuracy performance on the ROCF, and all parameters reflecting recognition ability on the TAVEC. The associations with the TMT parameters and reproduction performance on the ROCF were also seen in regression analysis. As a major source of emotional dysregulation, alexithymia has been implicated in somatosensory amplification, pain chronification and the pathogenesis of FMS (Wise and Mann, 1994; Huber et al., 2009; Hosoi et al., 2010; Castelli et al., 2012). In addition to FMS pain, alexithymia has been related to general distress, depression, anxiety and maladaptive coping, which are relevant therein (see Montoro et al., 2016 for an overview). Our data suggest that alexithymia may also contribute to the cognitive symptoms of FMS. Though theoretical models and empirical data concerning the linkage between alexithymia and performance are still scarce, it could be argued that cognitive impairments occur due to reduced resources being available to adjust emotional regulation, to in turn provide optimal processing conditions (Keefe et al., 2017). Though in the regression analysis in this study alexithymia emerged as an independent predictor of performance, distraction due to distress related to alexithymia may play an additional role.

Significant associations with attention, memory and planning also arose for pain catastrophizing. The corresponding CSQ scale score correlated positively with mistakes on the TMT and R-SAT, and with execution time during the number and letter sequence conditions of the TMT and the reproduction condition of the ROCF. Furthermore, higher levels of catastrophizing were associated with fewer correct responses and more mistakes on the TAVEC. Less accurate performance in the TMT, TAVEC and R-SAT in patients with high catastrophizing scores was also reflected in the regression analysis. Pain catastrophizing is recognized as a crucial factor in the development of chronic pain, especially in FMS (Geisser et al., 2003; Gracely et al., 2004). Catastrophizing may increase pain severity due to the inability to divert attention away from pain, and by enhancing pain-related negative affect (Crombez et al., 1998; Gracely et al., 2004). Furthermore, it has been argued that catastrophizing may significantly disrupt attentional processing. Crombez et al. (1998) reported that healthy individuals exhibiting high levels of pain catastrophizing experienced greater attentional interference due to electrocutaneous stimuli than those with low levels of catastrophizing. It may thus be hypothesized that FMS pain may more strongly affect cognition in patients displaying high catastrophizing due to greater disturbance of attention.

Finally, low levels of self-esteem were related to poorer attentional, memory and planning performance. The SS score correlated negatively with execution time during the visual scanning, number sequence, letter sequence and switching tasks of the TMT, as well as with error rates on this test. Higher values were also associated with faster performance during all conditions of the ROCF and ZMT, and with improved free recall and recognition performance on the TAVEC. A large proportion of these relationships was also seen in regression analysis. The association between self-esteem and cognition may be mediated by motivational factors. Self-esteem is related to self-confidence and self-efficacy expectation (Michielsen et al., 2006; Garaigordobil, 2013). These variables are considered important sources of achievement motivation that, in turn, may lead to higher effort during the performance of cognitive tasks, and thus better results (Zafra-Polo et al., 2014). Corroborating previous research, self-esteem was far lower in our FMS patients than in healthy individuals (Michielsen et al., 2006; Miró et al., 2011a; Garaigordobil, 2013; Peñacoba-Puente et al., 2015). Lower intrinsic motivation due to deficiencies in self-esteem may therefore have contributed to poor task performance.

One limitation of the study was the relatively small size of the control group, which may have limited the power of the statistical group comparison. Moreover, caution is required in the interpretation of the results of the exploratory correlation analysis considering the increased risk of alpha errors due to the high number of computed correlations. Regarding the selection of self-report scales, some relevant constructs may not have been taken into account. For example, emotional self-regulation, emotional competence or self-efficacy may be of interest in future studies. Finally, the conclusion of a causal contribution of affective factors to cognitive impairments in FMS cannot be drawn with certainty. By definition, the causal impact of cognitive deficits on affective variables, as well as mutual influences between cognition and emotion, also have to be considered.

To conclude, this study confirmed marked deficits in the domains of attention, memory and executive functions in FMS and revealed new knowledge regarding the psychological factors involved in their mediation. Overall, affect-related variables, i.e., positive and negative affect, alexithymia, pain catastrophizing and self-esteem, were as closely associated with neuropsychological test scores as pain severity. Our findings underline the importance of these variables in the genesis of cognitive symptoms in FMS. As an important factor of FMS pathology, emotional aberrances may also be relevant with respect to optimizing psychological therapy of FMS. Techniques addressing affect-related variables may constitute a helpful component of cognitive therapy in the management of FMS (Thieme and Turk, 2012; Duschek et al., 2013, 2014; Weiss et al., 2013). In addition to cognitive facilitation, these strategies may enhance the ability to cope with stress in everyday life, thereby helping to reduce pain and affective symptoms of the disease. It has been well-established that, for example, emotional skills training and mindfulness-based stress reduction exert beneficial effects on health and wellbeing, and reduce the burden of everyday stress (Slaski and Cartwright, 2003; Berking et al., 2008; Crowe et al., 2016; Saedpanah et al., 2016).

## Author Contributions

All authors contributed to the design of the study and to the analysis and interpretation of the data. CG-S conducted the experiment, acquired data, and drafted the first version of the manuscript. GRdP and SD critically revised and completed the manuscript and approved the submitted version.

## Conflict of Interest Statement

The authors declare that the research was conducted in the absence of any commercial or financial relationships that could be construed as a potential conflict of interest. The reviewer MA and handling Editor declared their shared affiliation.
